# Regulatory T cells: masterminds of immune equilibrium and future therapeutic innovations

**DOI:** 10.3389/fimmu.2024.1457189

**Published:** 2024-09-03

**Authors:** Junwei Ge, Xuan Yin, Lujun Chen

**Affiliations:** ^1^ Department of Tumor Biological Treatment, The Third Affiliated Hospital of Soochow University, Changzhou, Jiangsu, China; ^2^ Jiangsu Engineering Research Center for Tumor Immunotherapy, The Third Affiliated Hospital of Soochow University, Changzhou, Jiangsu, China; ^3^ Institute of Cell Therapy, The Third Affiliated Hospital of Soochow University, Changzhou, Jiangsu, China

**Keywords:** Treg, autoimmune diseases, cancer, immunotherapy, checkpoint inhibitors

## Abstract

Regulatory T cells (Tregs), a subset of CD4^+^T cells marked by the expression of the transcription factor forkhead box protein 3 (Foxp3), are pivotal in maintaining immune equilibrium and preventing autoimmunity. In our review, we addressed the functional distinctions between Foxp3^+^Tregs and other T cells, highlighting their roles in autoimmune diseases and cancer. We uncovered the dual nature of Tregs: they prevented autoimmune diseases by maintaining self-tolerance while contributing to tumor evasion by suppressing anti-tumor immunity. This study underscored the potential for targeted therapeutic strategies, such as enhancing Treg activity to restore balance in autoimmune diseases or depleting Foxp3^+^Tregs to augment anti-tumor immune responses in cancer. These insights laid the groundwork for future research and clinical applications, emphasizing the critical role of Foxp3^+^Tregs in immune regulation and the advancement of next-generation immunotherapies.

## 1 Introduction

Regulatory T cells (Tregs) are a unique group of lymphocytes within the immune system, playing vital roles in maintaining immune equilibrium, preventing autoimmunity, and reducing inflammation. Beyond their immune functions, research has shown that Tregs also aid in skeletal muscle repair via IL-33 and support scar healing by expressing Sparc (1). Tregs are primarily identified by the surface markers CD4 and the intracellular transcription factor forkhead box protein 3 (Foxp3), the latter being essential for their regulatory function (2). Additionally, they express specific molecules like PD-1 (programmed cell death protein 1) and CTLA-4 (cytotoxic T-lymphocyte antigen-4), which are key to their regulatory roles (3). Tregs regulate the immune response through both contact-dependent mechanisms (direct interaction with other immune cells) and contact-independent mechanisms, such as secreting inhibitory cytokines like IL-10, IL-35, and TGF-β (4).

Tregs protect the body from excessive inflammation and autoimmune damage by controlling the activation of other immune cells and moderating the intensity of immune responses. They can suppress self-reactive T cells and B cells, reducing attacks on self-tissues (5). Furthermore, the immunosuppressive factors they secrete inhibit inflammatory and autoimmune responses, and Tregs modulate the activity and function of other immune cells by inhibiting stimulatory molecules. In the tumor microenvironment (TME), Tregs play a multifaceted role. They mitigate excessive inflammation and tissue damage, potentially providing protective effects during tumor development. However, they also facilitate tumor cells in evading immune attacks, contributing to cancer progression (6). By suppressing immune cells such as CD4^+^T cells, CD8^+^T cells, natural killer (NK) cells, and dendritic cells (DCs), Tregs promote tumor growth and progression (7).

Our present review provides a detailed outlook of Treg characteristics, focusing on their high expression of molecular markers. It delves into the interactions between Tregs and other cells, highlighting the differences in Treg function and phenotype. Additionally, this review delves into the latest advancements in immunotherapy targeting Tregs, encompassing the development of targeted treatment strategies, innovative drug development, and clinical applications related to Treg signaling pathways.

## 2 Characterization and subtypes of Tregs

Tregs were first identified as CD4^+^T cells characterized by their elevated expression of CD25 (interleukin-2 receptor α-chain, IL-2Rα). This marker binds strongly to IL-2, enhancing Treg immune tolerance via the CD25/STAT5 signaling pathway (8). Additionally, studies have demonstrated that CD25^-^Tregs exhibit a similar memory/effector phenotype compared to CD25^+^Tregs, yet they maintain their suppressive capacity. Specifically, CD25^-^Tregs effectively inhibit the proliferation of effector T cells (T_eff_) and the production of cytokines *in vitro*, particularly in the context of HIV-Tuberculosis co-infection (9). The specific expression of Foxp3 in Tregs is a critical factor for their development, regulating inhibitory functions and defining Treg lineage identity (10). Mutations that inactivate Foxp3 can result in severe autoimmune disorders, such as X-linked immune dysregulation polyendocrinopathy enteropathy syndrome (IPEX), multiple endocrine neoplasia, and enteropathy (11). Interestingly, in human CD4^+^CD25^−^cells, TCR stimulation in the presence of TGF-β leads to a transient induction of Foxp3 without conferring a suppressive phenotype while similar cells in mice exhibit strong suppressive functions *in vitro* under the same conditions (12).

**Figure 1:**
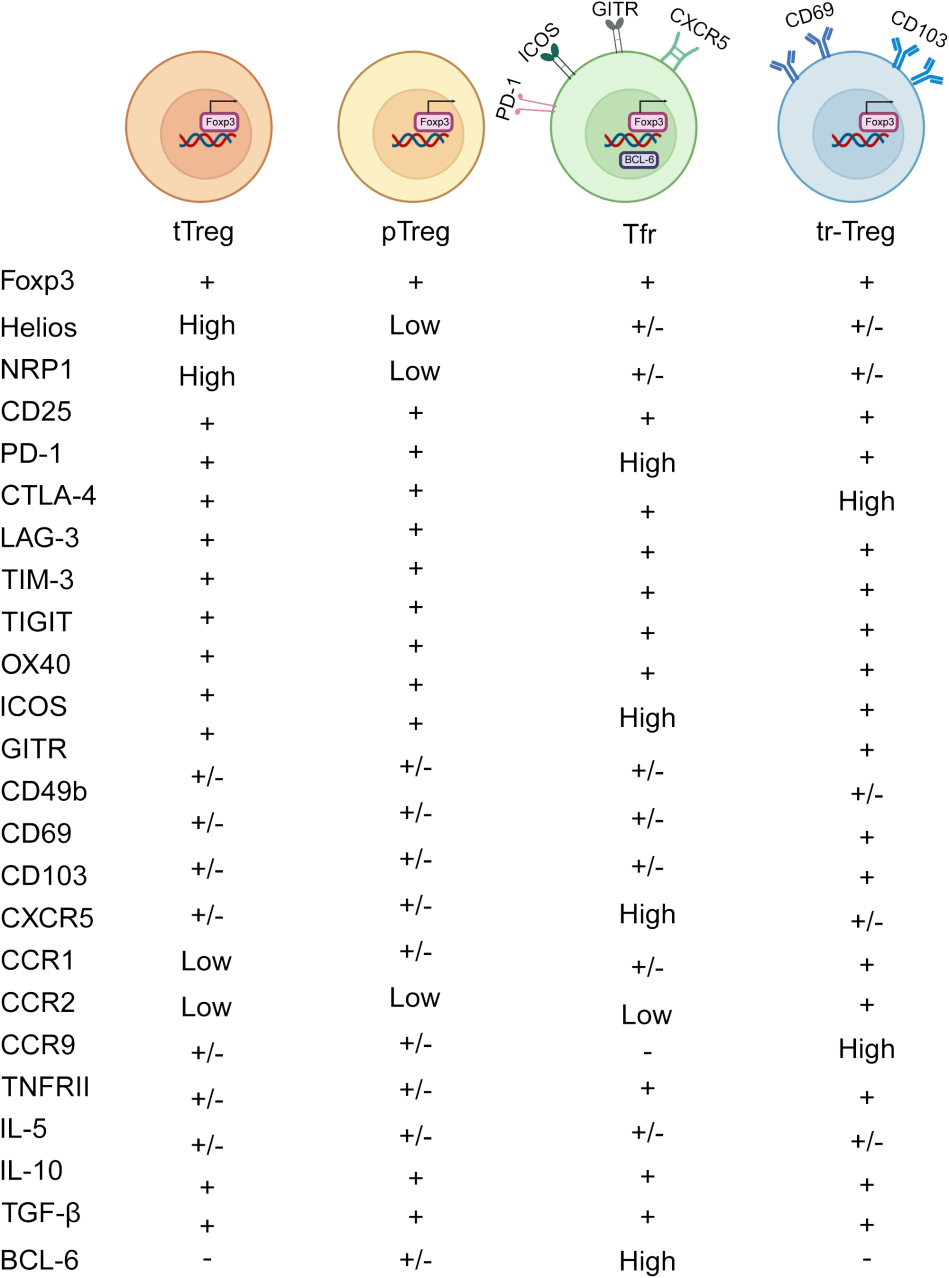
Heterogeneity of Treg subtypes. Treg cells comprise several subtypes based on their origin and function. tTregs are produced in the thymus, where they help maintain self-tolerance by inhibiting self-reactive T cells and facilitating central tolerance to self-antigens. pTregs are induced in peripheral lymphoid organs, mediating peripheral tolerance and regulating the intensity and scope of immune reactions. T_fr_ cells are enriched in lymph node follicles and splenic follicles, where they regulate B cell activation and antibody production within follicles, thus contributing to the regulation of humoral immune responses. Tr1 cells are distributed throughout peripheral tissues and lymphoid organs, producing abundant IL-10 and TGF-β to restrain immune cell activation and inflammation. tr-Tregs include subpopulations residing in tumor tissues, inflammatory tissues, and organ-specific tissues, regulating local immune responses and impacting tumor development, inflammation progression, and organ immune balance. CD103 and CD69 are classical markers of tr-Tregs, which may be upregulated in TI-Tregs.

CD25^+^Foxp3^+^Tregs, which develop and mature in the thymus, are known as thymic Tregs (tTregs) (13). Their development involves both T-cell receptor (TCR)-dependent and TCR-independent mechanisms. In the TCR-dependent pathway, thymic cells expressing TCR with intermediate affinity differentiate into CD4^+^CD25^+^Foxp3^-^Treg precursor cells. Meanwhile, the TCR-independent process relies on cytokines such as IL-2, IL-7, and IL-15 to regulate Foxp3 expression (14). Both precursor tTregs and mature Tregs display elevated expression of OX40, GITR, and TNFRII. When these molecules bind with their respective ligands, they drive the differentiation of thymic cells into tTregs (15). Furthermore, the *Kcnk18* gene encodes K2P18.1, which has been shown to promote Foxp3 expression via the NF-κB and NFAT pathways, thereby supporting the development and maturation of tTregs (16) (**Figure 1**).

Naïve CD4^+^T cells, when encountering antigen stimulation in peripheral tissues, can become peripheral Tregs (pTregs), especially under the influence of factors like IL-2, TGF-β, and retinoic acid (17). These pTregs primarily inhibit reactions against exogenous antigens, orchestrating tolerance at the periphery and maintaining tissue equilibrium. While neuropilin-1 (Nrp1) and Helios have been suggested as markers to distinguish tTregs from pTregs, it’s important to recognize that pTregs and inducible Tregs (iTregs) can also express Nrp1. This is because Nrp1 expression is positively regulated by TGF-β, leading TGF-β-induced iTregs to express Nrp1 as well (18). Furthermore, decreased expression of Nrp1 can be induced by IFN-γ, thereby impairing the activity and stability of Treg (19). Foxp3^+^Tregs generated by stimulating conventional T cells (T_conv_) in the presence of TGF-β are referred to as iTregs. Compared to the more stable pTregs, iTregs may represent a less stable cell population with potential therapeutic applications in preventing autoimmune diseases and transplant rejection, primarily by inhibiting T cell activation (20). However, detailed studies on the mechanisms of iTreg action remain limited (**Figure 1**).

Follicular regulatory T cells (Tfr) are another subset within the Treg family, characterized by high levels of PD-1, GITR, ICOS, CXCR5, and BCL6 (21). Tfr cells play a crucial role in suppressing the differentiation of B cells into plasma cells, with neuritin enhancing BCL6 expression in Tfr cells to prevent this transition (22) (**Figure 1**).

Tissue-resident Tregs (tr-Tregs) are distributed across various non-lymphoid organs, such as the skin, lungs, liver, adipose tissue, skeletal muscle, small intestine, and large intestine (23). These tr-Tregs are pivotal in tissue repair and maintaining homeostasis, characterized by CD103 and CD69 (24). CD103, known as α_E_ integrin facilitates the retention of lymphocytes within epithelial tissues and act as a receptor for E-cadherin. In different tissues, tr-Tregs perform indispensable functions. For instance, skin-resident Tregs are integral to wound healing and the regeneration of hair follicle stem cells (HFSCs) (25). Meanwhile, skeletal muscle-resident Tregs secrete amphiregulin (Areg), which promotes satellite cell differentiation and enhances muscle repair by inhibiting T_conv_ (26). Tr-Tregs are vital in the TME for promoting angiogenesis by releasing vascular endothelial growth factor (VEGF) and secreting higher levels of VEGF-A under hypoxic conditions, thereby supporting tumor growth and metastasis (27). Tr-Tregs exhibit increased expression of chemokine receptors such as CCR1, CCR2, and CCR9, along with immune checkpoint molecules like TIM-3, GITR, and CD39 (**Figure 1**).

## 3 Interaction between Tregs and other cells

### 3.1 Interaction between Tregs and DCs

DCs, vital immune sentinels found in nearly all peripheral tissues, play crucial roles in immune regulation and are primarily classified into three types: conventional DCs (cDCs), plasmacytoid DCs (pDCs), and monocyte-derived DCs (MoDCs), with cDCs subdivided into type 1 (cDC1) and type 2 (cDC2) (28). In immune responses, cDC1 cells primarily stimulate CD8^+^T cells and induce Th1 cell reactions by cross-presenting exogenous antigens, playing key roles in antiviral and antitumor immunity. In contrast, cDC2 cells activate CD4^+^T cells, contributing to inflammatory responses and immune regulation (29).

DCs activate naïve Tregs by providing three essential signals: presenting exogenous antigens via major histocompatibility complex (MHC) molecules, delivering co-stimulatory molecules (such as CD80 and CD86), and releasing cytokines (including TGF-β) to facilitate Treg development. Furthermore, DCs induce tolerance by expressing the immunoregulatory enzyme indoleamine 2,3-dioxygenase 1 (IDO1), maintaining self-antigen tolerance (30). Additionally, epithelial cells from the intestinal lining contribute to the generation and function of tolerogenic CD103^+^DCs, which could help regulate Tregs.

**Figure 2:**
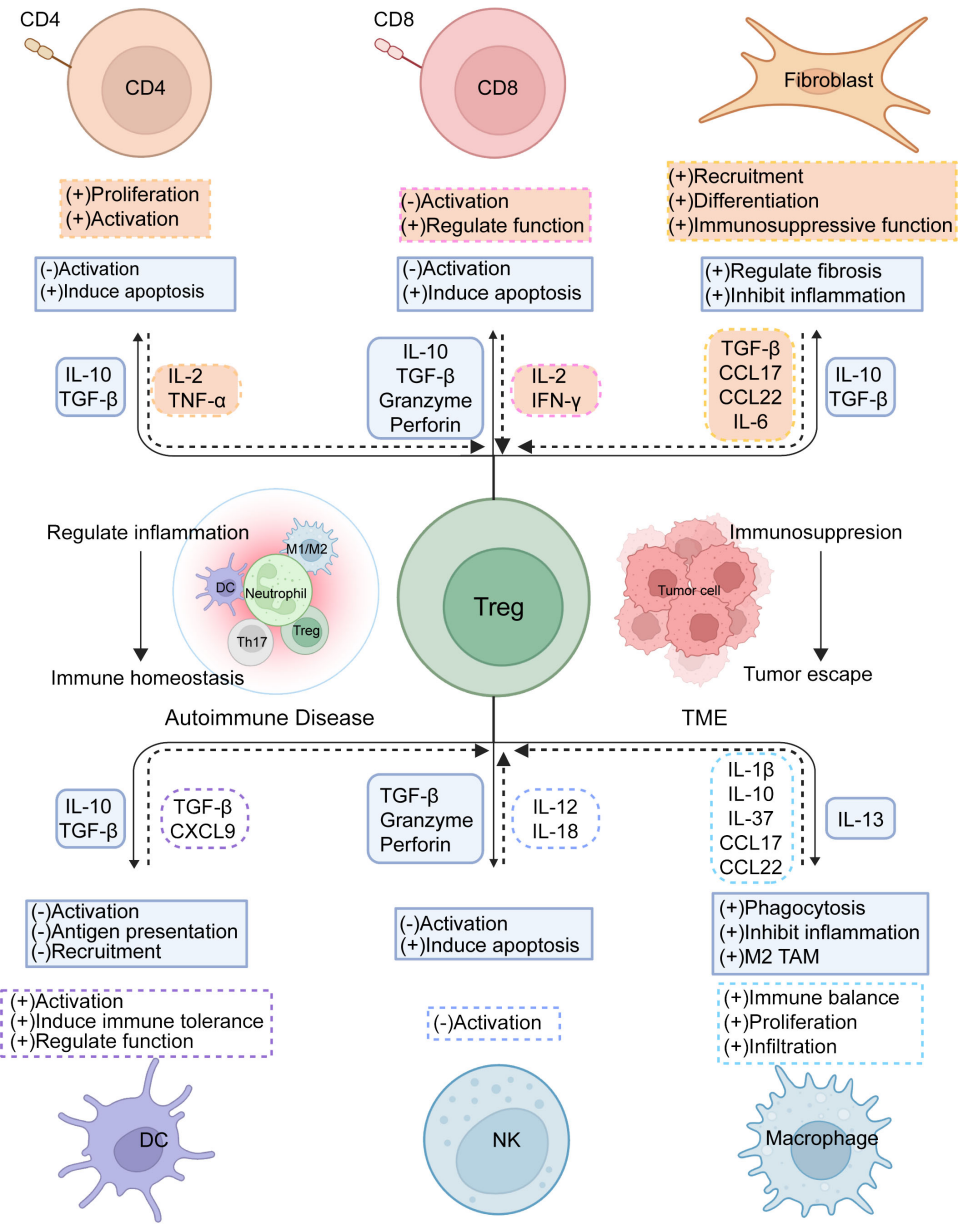
Tregs immunoregulation. Treg cells interact with diverse cells in the immune microenvironment to regulate immune stability and foster suppression within the TME. By expressing suppressive receptors and releasing inhibitory cytokines, Treg cells modulate the activation and differentiation of T_eff_ cells, helping to restrain excessive immune reactions and prevent autoimmune conditions, while also governing immune cell-driven tumor control. Concurrently, other immune cells support Treg activation, proliferation, and functionality, collectively maintaining immune equilibrium.

Tregs critically influence DCs by producing IL-10 and TGF-β to regulate DC initiation and function, and by limiting the recruitment of CCR5-dependent DCs in lymph nodes through the inhibition of CCR5 ligand production (31). Additionally, Tregs block IL-15Rα exposure on DCs, affecting DC-mediated NK cell proliferation, a regulatory mechanism particularly significant in autoimmune diseases as it controls DC/T cell autoreactivity triggered by NK cells (32). The interplay between Tregs and DCs is also crucial in anti-tumor immunity. Tumor-infiltrating Tregs (TI-Tregs) receive TCR signals from cDCs and downregulate CD80/CD86 expression on cDCs *via* CTLA-4, thereby inhibiting T cell activation and expansion (33). Furthermore, Tregs expressing CXCR3 co-localize with DCs producing CXCL9 in tumors, which affects the presentation of tumor-derived antigens and dampens the activity of anti-tumor CD8^+^T cells (**Figure 2**).

### 3.2 Interplay between Tregs and NK cells

NK cells utilize a variety of pathways to eliminate pathogens and tumor cells, including granule enzyme and perforin pathways, as well as death receptor-mediated pathways. They also secrete chemokines and cytokines, playing a vital role in immune regulation. However, these functions can be partially suppressed by Tregs. Tregs modulate NK cell activity through several mechanisms. For instance, they produce TGF-β, which suppresses NKG2D expression and weakens NK cell responses (34). Additionally, Tregs can trigger NK cell apoptosis via a mechanism dependent on granzyme B and perforin, thus hindering tumor clearance (35). Another pathway involves inhibiting IL-2-induced NK cell activation by depriving NK cells of IL-2 signaling. Furthermore, IL-37 produced by Tregs can interact with the inhibitory receptor IL-1R8 on NK cells, causing phenotypic changes and reduced functionality (36).

Notably, NK cells also exert a significant influence on Treg activity (36). For instance, in type 1 diabetes, the upregulation of IL-12/IL-18 allows NK cells to enhance CD25 expression, directly competing with Tregs for IL-2, which reduces Treg suppression capability (37). In-depth research into the interactions between NK cells and Tregs can offer valuable insights into the regulatory mechanisms of the immune system. Understanding these dynamics could lead to the development of new therapeutic strategies to enhance immune responses against pathogens and tumors (**Figure 2**).

### 3.3 Interaction between Tregs and T lymphocytes

Tregs regulate antigen-presenting cells (APCs), primarily DCs, through various mechanisms, directly or indirectly suppressing T_eff_. These regulatory mechanisms involve the release of inhibitory cytokines and the expression of inhibitory receptors. While much research has focused on how Tregs inhibit CD4^+^T cells, they also modulate CD8^+^T cells, which are crucial effector cells in antitumor immune responses. CD8^+^T cells become activated by recognizing antigen peptides presented on MHC-I molecules and mature into cytotoxic effector T cells, leading to the death of target cells (38).

Tregs can release granzyme B and perforin to trigger apoptosis in CD8^+^T cells in the TME (35). In maintaining immune homeostasis, Tregs curb the expansion of CD8^+^T cells and prevent the spontaneous transition of memory CD8^+^T cells into effector cells (39). Additionally, Tregs mitigate the response of CD8^+^T cells to antigens. They employ multiple mechanisms to suppress CD8^+^T cell activity. For instance, the CTLA-4 molecule on Tregs binds to the B7 molecule on APCs, reducing CD8^+^T cell activation. Moreover, Tregs secrete inhibitory cytokines like IL-10 and TGF-β, which dampen CD8^+^T cell activity and function. These cytokines can impede APC activity or directly affect CD8^+^T cells, diminishing their antigen sensitivity, inhibiting cytokine production, and inducing apoptosis.

Furthermore, Tregs restrict the availability of IL-2 to suppress CD8^+^T cell activity and prevent APCs from producing chemokines CCL3, CCL4, and CCL5, thereby curtailing CD8^+^T cell migration and activation (40) (**Figure 2**). Gaining deeper insights into how Tregs suppress CD8^+^T cells can enhance our comprehension of immune regulation in both immune tolerance and diseases. This knowledge lays a crucial theoretical foundation for developing treatments for related disorders.

### 3.4 Interaction between Tregs and macrophages

Macrophages, originating from bone marrow, are widely distributed in various tissues, including the liver, lungs, skin, and lymph nodes. These immune cells have the capacity to detect, ingest, and decompose pathogens, playing a significant role in immune responses by demonstrating both pro-inflammatory and anti-inflammatory properties (41). Moreover, macrophages are involved in antigen presentation and contribute to the eradication of tumor cells. They are categorized into M1 macrophages, which are pro-inflammatory and secrete cytokines such as IL-1β, IL-6, IL-12, IL-23, and TNF-α, and M2 macrophages, which possess anti-inflammatory and immunoregulatory functions, producing cytokines like IL-10 and TGF-β (42).

An intricate network of interactions exists between macrophages and Tregs, playing essential roles in immune regulation. Tregs release IL-13, which induces macrophages to produce IL-10, enhancing the phagocytosis of apoptotic cells via a Vav1-Rac1-mediated mechanism (43). Additionally, LAG-3 on Tregs inhibits the CD40 and NF-κB signaling pathways in CX3CR1^+^macrophages, reducing the secretion of IL-1β and IL-23. This reduction indirectly affects innate lymphoid cells (ILC3) production, thereby regulating intestinal inflammation and maintaining intestinal homeostasis (44). Conversely, IL-1β produced by intestinal macrophages acts on ILC3s within the intestine, which subsequently generate granulocyte-macrophage colony-stimulating factor (GM-CSF) that induces myeloid cells, including DCs and macrophages, to produce retinoic acid and IL-10, thereby supporting the transformation and proliferation of Tregs and maintaining immune balance in the intestine (45). Additionally, the transcriptional co-repressors TLE3 and TLE4 in intestinal macrophages negatively regulate MMP9 transcription, inhibiting potential TGF-β activation and thus limiting the expansion of Tregs and Th17 cells (46).

The interplay between macrophages and Tregs within the TME is highly complex. Tregs influence macrophage function and polarization, affecting tumor progression. For example, Tregs indirectly enhance M2-type tumor-associated macrophages (TAMs) by preventing the release of IFN-γ from CD8^+^T cells, increasing the population of TAMs and supporting their activity (47). Moreover, the expression of LAG-3 and TIM-3 on Tregs prompts macrophages to produce IL-10, which lowers the levels of MHCII, CD80, CD86, and TNF-α, thereby modulating the pro-inflammatory activation of macrophages. Conversely, macrophages primarily support Treg cell proliferation and function, promoting their infiltration and activity within the TME. For instance, macrophages can stimulate Treg cell proliferation and induce IL-10 production by releasing inhibitory factors such as IL-37, which dampens the activation of cytotoxic T cells and NK cells, reducing their attacks on tumor cells (48). Chemokines secreted by macrophages, like CCL17 and CCL22, attract Tregs to the tumor area, enhancing Treg infiltration within the TME and further sustaining the tumor’s ability to evade immune detection (49). TAM-derived CXCL1 promotes the conversion of naive CD4^+^T cells into Tregs by activating the NF-κB/Foxp3 signaling pathway at the transcriptional level, fostering an immunosuppressive environment (50). Further exploration of these regulatory mechanisms and interaction modes can aid in developing more effective tumor immunotherapy strategies, leading to better treatment outcomes for cancer patients (**Figure 2**).

### 3.5 Interaction between Tregs and fibroblasts

Fibroblasts, essential mature connective tissue cells derived from mesenchymal origins, play significant roles in wound healing, extracellular matrix (ECM) remodeling, inflammation, and cancer progression (51). In rheumatoid arthritis (RA), fibroblasts activated by tumor necrosis factor (TNF) produce high levels of chemokines such as IL-6, CXCL12, and CCL2, which enhance the recruitment and attraction of monocytes. Similarly, in inflammatory bowel disease (IBD), fibroblasts contribute to inflammation through the secretion of factors like IL-11, IL-24, and IL-13RA2. These observations highlight the critical role of fibroblasts in both inflammation and fibrosis (52).

In the TME, normal fibroblasts act as barriers to tumor development, while cancer-associated fibroblasts (CAFs) enhance tumor characteristics, such as cancer cell growth and invasion, angiogenesis, and ECM remodeling. CAFs are classified into three types: myCAF (resembling myofibroblasts), iCAF (with inflammatory properties), and apCAF (with antigen-presenting features) (53). CAFs interact with Tregs through various mechanisms, collectively promoting tumor immune tolerance and tumorigenesis. They affect Treg activity and function by releasing different signaling molecules. Notably, TGF-β from CAFs has a dual role: it promotes CXCL13 secretion from CD4^+^T and CD8^+^T cells while decreasing IL-2 levels, leading to an increase in Treg populations, and additionally encourages the formation of the myCAF phenotype by downregulating IL1R1 expression (54). Chemokines CCL17 and CCL22 released by CAFs attract Tregs, further intensifying immune suppression. Additionally, CAFs trigger signal transducer and activator of transcription 3 (STAT-3) activation through IL-6 production, which recruits immunosuppressive IDO^+^DCs and inhibits T-cell proliferation while promoting Treg production (55). It has been observed that IL-6 release by CAFs induces the differentiation of CD73^+^γδ Tregs, which secrete more adenosine, thus enhancing immune suppression (56). Furthermore, INHBA/recombinant activin A in CAFs induces PD-L1 expression via an autocrine mechanism involving SMAD2-dependent signaling, promoting Treg differentiation (57). Activation by the transcription factor Snail1 also enhances interactions between CAFs and macrophages, increasing Treg activity (58). Our recent work has also indicated that Tregs promote MHC-II expression on CAFs by expressing IL1R2, thus facilitating Treg accumulation and manifesting Treg function (59) (**Figure 2**).

## 4 Tregs in autoimmune diseases/cancer

### 4.1 Tregs in autoimmune diseases

**Figure 3:**
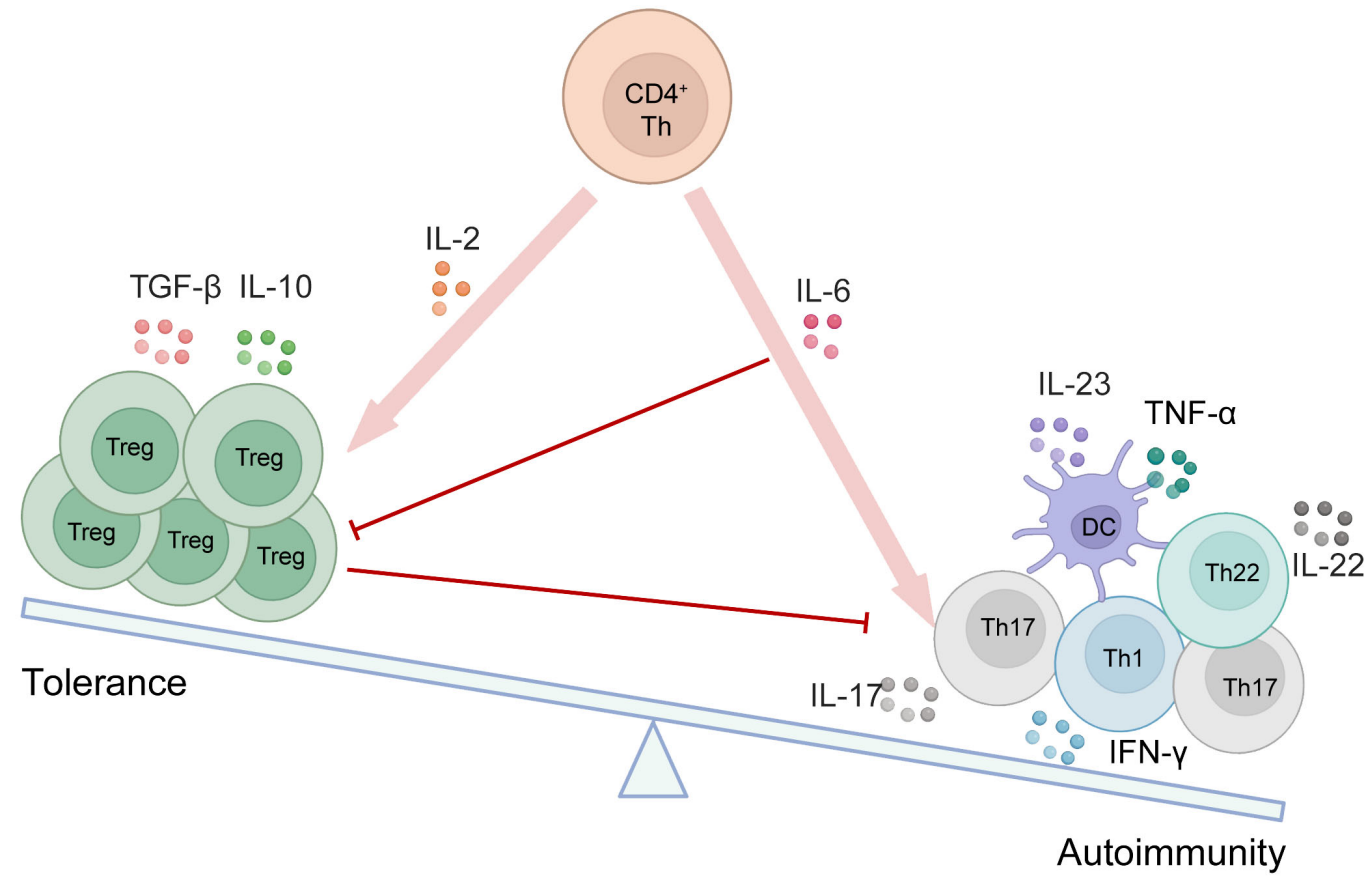
The equilibrium between Tregs and other immune cells is disrupted in autoimmune disorders. As depicted in the schematic, various cytokines contribute to the initiation and generation of different cell types. Red arrows indicate promotion, while red lines represent inhibition.

In autoimmune diseases, Tregs are crucial for regulating the inflammatory process. Their dysregulation is associated with several autoimmune conditions, including multiple sclerosis (MS), psoriasis, systemic lupus erythematosus (SLE), IBD such as Crohn’s disease and ulcerative colitis, and RA. Thus, maintaining the appropriate numbers and activity of Tregs is vital for sustaining normal host immunity and overall health. Key examples of Treg imbalance in patients will be explored in various autoimmune manifestations as below (**Figure 3**).

#### 4.1.1 MS

MS is an autoimmune disease characterized by T cell-mediated demyelination and neurodegeneration within the central nervous system (CNS). Experimental autoimmune encephalomyelitis (EAE) in mice is the primary animal model used to study MS (60). Although the exact etiology of MS remains elusive, Th17 cells, which secrete IL-17 and IL-22, and Tregs, which produce IL-10 and TGF-β, are critical in the disease pathogenesis and in the EAE model. In MS patients, both the number and function of Tregs in the cerebrospinal fluid are diminished (61). IL-17 and IL-22 contribute to the disruption of the blood-brain barrier and facilitate the recruitment of CD4^+^T cells and neutrophils to the CNS, while IFN-γ primarily enhances macrophage recruitment (62). Tregs counteract Th17 cells throughout the course of MS by inhibiting self-reactive Th1 and Th17 cells. Furthermore, during the remission phase in EAE mice, Tregs accumulate in the CNS, with IL-10 playing a pivotal role in this process (63).

CD39, also known as nucleoside triphosphate diphosphohydrolase 1 (NTPDase1), is primarily responsible for breaking down extracellular ATP and ADP into AMP, which is subsequently converted into adenosine by CD73 (64). Adenosine has anti-inflammatory and immunomodulatory effects, making CD39 a crucial factor in regulating the immune response. Studies have shown that CD39’s regulatory function enhances the migratory behavior of both CD4^+^T cells and Tregs (including CCR5^+^Tregs and CCR6^+^Tregs) in the CNS and lymphatic drainage sites of EAE mice and facilitates their migration *in vitro*. Lack of CD39 eliminates the accumulation of Tregs in EAE and correlates with heightened Th1/Th17 signaling in the CNS and gut-associated lymphoid tissues (65).

IL-6 is a pivotal cytokine in promoting Th17 differentiation, and the IL-6 signaling pathway, along with STAT-3, plays a crucial role in inhibiting the development of iTregs. Consequently, abnormal activation of the IL-6/STAT-3 pathway can lead to reduced Treg numbers and function, disrupting the balance between T_eff_ cells and Tregs. This imbalance exacerbates the onset and progression of autoimmune diseases (66).

#### 4.1.2 Psoriasis

Psoriasis is a chronic autoimmune disorder characterized by the increased proliferation of skin keratinocytes, resulting in epidermal thickening and scaling (67). Clinically, psoriasis often manifests as erythematous plaques, scales, and skin inflammation, primarily caused by the accumulation of inflammatory cells, including CD4^+^T cells, CD8^+^T cells, NK cells, and DCs. The development of psoriasis is closely linked to the abnormal activation of the immune system, particularly involving the dysfunction of various T-cell subpopulations (68). This dysregulated activation leads to inflammatory responses and skin lesions, with pro-inflammatory cytokines such as IFN-γ, TNF-α, and IL-17 being secreted by cells like Th1, Th17, and Th22. DCs also play a crucial role in psoriasis by excessively releasing inflammatory mediators like IL-23 and TNF-α. The IL-23/IL-17 axis is recognized as a major immune pathway involved in the development of psoriasis (69).

Tregs play a crucial role in suppressing the activation of other immune cells and preventing autoimmune reactions, thereby maintaining immune homeostasis. However, research indicates that both the number and functionality of Tregs are often impaired in patients with psoriasis (70). This impairment can lead to a loss of the immune system’s regulatory function against self-tissues, triggering excessive inflammatory and immune responses, which further promote the development of psoriasis. Additionally, studies have shown a notable increase in γδT cell numbers in psoriatic lesions, which release pro-inflammatory cytokines like IL-17, potentially contributing to the formation and progression of skin lesions (71).

Izumo1R is a folate receptor present on CD4^+^T cells, particularly on Tregs. Although the precise role of Tregs in psoriasis requires further exploration, studies using a mouse model with Treg-specific Izumo1r deficiency have shown no significant impact on Treg development and homeostasis. However, this deficiency results in immune dysregulation in the skin, accompanied by the dysregulation of γδT cells, underscoring the importance of Izumo1R expression on Tregs in the development of psoriasis (72).

#### 4.1.3 Other autoimmune diseases

Tregs also play a crucial role in various autoimmune diseases such as SLE, IBD, and RA. As immune regulatory cells, Tregs help maintain the balance of inflammatory responses through their unique immunomodulatory effects. In SLE, excessive expression of type I interferons (IFN-I) is a hallmark feature. IFN-I signaling affects the interplay between activated Tregs and T_eff_, compromising the regulatory role of Tregs in viral infections and within the TME. Research has identified an exhausted phenotype of Tregs in SLE patients, specifically CCR7^lo^CD74^hi^ Tregs. IFN-I signaling significantly contributes to the development of Treg cell exhaustion and functional impairments. Similarly, CD4^+^T-cell data from ulcerative colitis patients show analogous characteristics of Treg dysfunction (73). In IBD patients, increased levels of HLA-DR^+^CD38^+^T cells, including IL17A^+^Tregs that produce inflammatory cytokines, are observed in colon mucosal samples. Patients with Crohn’s disease exhibit elevated levels of IL1B^+^Tregs in peripheral blood mononuclear cells (74). Regarding RA, a diminished expression of EZH2 in CD4^+^T cells of affected individuals leads to the down-regulation of RUNX1 and the up-regulation of SMAD7, which in turn inhibits Foxp3 transcription and suppresses the differentiation of Tregs (75). In summary, Tregs are critically important across various autoimmune diseases, and their dysfunction can lead to immune imbalance and disease progression.

### 4.2 Immunotherapeutic strategy targeting Tregs in autoimmune diseases

Enhancing the immune system’s regulatory function is crucial for treating autoimmune conditions. Current strategies primarily focus on boosting Treg activity, which is vital for suppressing harmful immune responses (76). One promising approach involves targeting IL-2, a molecule essential for the growth and function of various immune cells, including Tregs. In a Phase I/II trial, researchers have identified the optimal dose of IL-2 for safely expanding Tregs in children with Type 1 Diabetes (T1D) (NCT01862120) (77). Another study has revealed that administering a low dose of IL-2 per day selectively activates and expands Tregs in patients with systemic sclerosis (SSc) without activating T_effs_, paving the groundwork for a Phase II efficacy trial (NCT01988506) (78). Additionally, rapamycin has been shown to selectively expand Tregs while maintaining their suppressive phenotype and function, as demonstrated in a mouse model of T1D (79). Furthermore, the high expression of TNFR2 on Tregs can selectively eliminate autoreactive CD8^+^T cells, suggesting its potential benefits in treating autoimmune diseases (80). Despite its potential, administering low doses of IL-2 carries risks, including off-target effects due to effector cell activation and a short lifespan in the body. IL-2 muteins are engineered to overcome the limitations of conventional IL-2 therapies. By decreasing IL-2’s affinity for specific receptor subunits, such as CD122 or CD25, these modified proteins alter IL-2 signaling properties, thereby reducing T_eff_ activation and enhancing Tregs selectivity (81). A novel IL-2 mutein has demonstrated significant efficacy in preclinical models by increasing Treg numbers and enhancing their suppressive activity, which extends the survival of allogeneic grafts and promotes antigen-specific tolerance (82). Additionally, compared to wild-type IL-2, these muteins have shown greater selectivity and efficacy in disease control within non-obese diabetic (NOD) models (81).

PolyTreg therapy which involves the isolation, *in vitro* expansion, and reinfusion of a patient’s own Tregs, designed to enhance both the number and function of Tregs. This technique is currently being tested in clinical trials for T1D (83). Additionally, combining polyTregs with low-dose IL-2 may further augment Treg numbers and functionality. A Phase I study indicated that this combination therapy could expand Tregs and cytotoxic T lymphocytes (CTLs) in T1D patients, with a favorable safety profile (NCT01210664, NCT02772679) (84). These findings suggest that the combination of Tregs with other therapies holds significant potential for treating autoimmune diseases, though further investigation and validation are necessary (**Table 1**).

**Table 1:** Clinical trials to target Tregs to treat autoimmune diseases.

Therapies	Diseases	Phase	Study ID
LD IL-2	SLE	Phase 2	NCT02465580
	SLE	Phase 2	NCT02955615
	Remitting MS	Phase 2	NCT02424396
	HCV-induced vasculitis	Phase 1/2	NCT00574652
	pSS	Phase 2	NCT02464319
	SSc	Phase 1/2	NCT01988506
	T1D	Phase 1/2	NCT01862120
PolyTreg	Crohn’s Disease	Phase 1/2	NCT03185000
	Cutaneous lupus	Phase 1	NCT02428309
	T1D	Phase 1	NCT01210664
PolyTreg+ LD IL-2	T1D	Phase 1	NCT01210664
	T1D	Phase 1	NCT02772679

LD IL-2, Low dose IL-2.

Another approach involves Treg-cell therapy based on genetic engineering to enhance Treg-cell function and redirect their targeting specificity, such as transgenic TCR therapy and chimeric antigen receptor (CAR) therapy (85). Since Tregs and self-reactive T cells possess a similar TCR repertoire, introducing a transgenic TCR into Tregs can efficiently direct them toward specific antigens of interest (86). However, the safety and efficacy of Tregs engineered with transgenic TCRs are still under investigation. Compared to TCR Tregs, CAR Tregs offer several advantages, including maintaining the phenotype and function of Tregs, retaining the ability to expand to therapeutic levels, and minimizing cytotoxicity to target cells. Additionally, CAR Tregs operate independently of the MHC and have a lower dependence on IL-2.

Autoimmune diseases are characterized by abnormalities of immune responses and inflammation, yet effective cures remain elusive. While Treg therapy shows promise in modulating the immune system, its efficacy is limited, necessitating further in-depth research. Currently, Treg therapy faces several challenges in treating autoimmune diseases, including unstable Treg cell function and inconsistent treatment outcomes. To overcome these challenges, it is essential to bolster foundational research, explore innovative treatment strategies, and conduct additional clinical trials to identify more effective therapies.

### 4.3 Tregs in cancer

TI-Tregs represent a critical mechanism for cancer evasion, posing a significant challenge to tumor-immune defenses and immunotherapy. In various cancers, such as liver cancer (87), gastric cancer (88), and bladder cancer (89), the presence of TI-Tregs often correlates with poor prognoses. The ratio of Tregs to CD8^+^T cells within tumors serves as a crucial prognostic factor, typically indicating unfavorable outcomes when skewed towards a higher proportion of Tregs (89). However, the role of Tregs in colorectal cancer (CRC) remains controversial, partly due to the diverse functions and phenotypes of Tregs (90).

Tregs can include both suppressive and non-inhibitory subsets. For example, peripheral blood Tregs can be divided into three main subsets based on the expression levels of Foxp3 and CD45RA (91), namely (1) Fr.I: Foxp3^lo^CD45RA^+^, known as naive Tregs (nTregs); (2) Fr.II: Foxp3^hi^CD45RA^-^, known as effector Tregs (eTregs), characterized by high CTLA-4 expression and strong immunosuppressive functions; (3) Fr.III: Foxp3^lo^CD45RA^-^, which primarily release inflammatory cytokines like IL-2 and IFN-γ.

The proportions of these different subsets in tumors can have varying impacts on prognosis. In most cancers, highly CTLA-4-expressing Fr.II eTregs are the predominant tumor-infiltrating Foxp3^+^Tregs. Saito et al. have further classified CRC into two types based on the frequency of Fr.III cells: Type A and Type B. Compared to Type B CRC, Type A CRC has fewer eTregs overall but higher *in vitro* suppressive activity, leading to a poor prognosis (92). Type B CRC has a higher frequency of Fr.III cells secreting IL-17 and IFN-γ, possibly originating from non-Tregs stimulated by IL-12 and TGF-β (93). These findings underscore the importance of assessing Treg subset distribution in tumor tissues (**Table 2**).

**Table 2:** Functional classification of human Tregs in the blood and tumors.

Peripheral blood				Tumor	
				Type A	Type B
Subset	Fr.I: nTreg	Fr.II: eTreg	Fr.III: non-Treg	Fr.II: eTreg	Fr.II: eTreg + Fr.III: non-Treg
Marker	Foxp3^lo^CD45RA^+^/CD25^lo^	Foxp3^hi^CD45RA^-^/CD25^hi^	Foxp3^lo^CD45RA^-^/CD25^lo^	―	―
Characteristics	Weak immunosuppressive activity	Strong immunosuppressive activity	No immunosuppressive activity	Low ratio of non-Treg	High quantity of Tregs
	Express naïve cell markers such as CCR7 and CD62L	Produce immunosuppressive cytokines (IL-10 and TGFβ)	Produce immunostimulatory cytokines (IL-2、IFN-γ)	Strong immunosuppressive activity in eTreg	Low expression of CTLA-4/High expression of IL-17 in non-Treg
	Differentiate to eTreg cells upon TCR stimulation	Prone to apoptosis	Heterogeneous population		

nTreg, naïve Treg; eTreg, effector Treg.

### 4.4 Immunotherapy for targeting Tregs in cancer

Research has shown that tr-Tregs exhibit diverse gene expression patterns similar to TI-Tregs. However, the differences between tr-Tregs and Tregs in normal tissue reflect the unique physiological characteristics of the TME, suggesting that tr-Tregs may have distinct functional and survival requirements (27). The increase and heightened activity of Tregs inhibit the immune system’s ability to attack tumors, thereby providing favorable conditions for tumor escape. Furthermore, by suppressing the function of T cells specific to tumor antigens, Tregs exacerbate immune tolerance, hindering the effectiveness of immunotherapy. Consequently, targeting Tregs has emerged as a crucial strategy to rejuvenate the immune system’s capacity to recognize and eliminate tumors (**Table 3**).

**Table 3:** Clinical trials to target Tregs in cancer patients.

Targets	Drugs	Tumor types and combinational therapies	Phase	NCT number
Foxp3	AZD8701	NSCLC	Phase 1	NCT04504669
CD25	Basiliximab	Glioblastoma Multiforme	Phase 1	NCT00626483
	Daclizumab	Melanoma	Phase 1/2	NCT00847106
PD-1	Pembrolizumab	NSCLC	Phase 2	NCT02343952
		Urothelial Cancer	Phase 2	NCT02335424
		Melanoma	Phase 2	NCT03897881
	Nivolumab	Advanced Melanoma + All-Trans Retinoic Acid	Phase 1/2	NCT03200847
		Metastatic SCLC	Phase 1/2	NCT01928394
		Stomach Cancer	Phase 3	NCT02872116
PD-L1	Atezolizumab	Advanced NSCLC	Phase 2	NCT02848651
	Avelumab	Bladder Cancer	Phase 1/2	NCT03498196
	Durvalumab	Head and Neck Cancer + Cetuximab	Phase 2	NCT03691714
CTLA-4	Ipilimumab	Pancreatic Cancer	Phase 2	NCT00112580
		Hepatocellular Carcinoma + Nivolumab	Phase 2	NCT03222076
		Peritoneal Mesothelioma + Nivolumab	Phase 2	NCT05041062
	Tremelimumab	Endometrial Cancer + Durvalumab	Phase 2	NCT03015129
		Prostate Cancer + Durvalumab	Phase 2	NCT02788773
		Urothelial Bladder Cancer	Phase 2	NCT02527434
LAG-3	Relatlimab	Melanoma + Nivolumab	Phase 2/3	NCT03470922
	MK-4280	Advanced colorectal cancer + pembrolizumab	Phase 1	NCT02720068
TIM-3	Sabatolimab	Advanced Malignancies + Spartalizumab	Phase 1/2	NCT02608268
	Sym023	Solid Tumors	Phase 1	NCT03489343
TIGIT	Tiragolumab	NSCLC + Atezolizumab	Phase 2	NCT03563716
OX40	INCAGN01949	Metastatic Cancer	Phase 1/2	NCT02923349
GITR	TRX518	Solid Tumors + Nivolumab	Phase 1	NCT02628574
		Unresectable Malignant Melanoma	Phase 1	NCT01239134
ICOS	Vopratelimab	Advanced solid tumors	Phase 1/2	NCT02904226
TGF-β	Fresolimumab	Metastatic Breast Cancer	Phase 2	NCT01401062
TGF-βR	Vactosertib	Stomach Cancer + Durvalumab	Phase 2	NCT04893252
		Metastatic Colorectal Cancer + Pembrolizumab	Phase 1/2	NCT03724851
CCR4	KW-0761	ATL/PTCL	Phase 1	NCT00355472
	Mogamulizumab	Solid Tumors + Nivolumab	Phase 1/2	NCT02705105
CCR8	GS-1811	Advanced solid tumors + Zimberelimab	Phase 1	NCT05007782
PI3Kα	Alpelisib	Breast Cancer + Fulvestrant	Phase 3	NCT02437318
		TNBC	Phase 1	NCT01623349
PI3Kδ	Idelalisib	Chronic Lymphocytic Leukemia	Phase 3	NCT01539512
PI3Kδ/γ	gedatolisib	Breast Cancer	Phase 1	NCT02684032
mTOR	Everolimus	Breast Cancer	Phase 3	NCT00863655
	Everolimus	Pancreatic Cancer	Phase 2	NCT00409292
IDO	GDC-0919	Solid Tumors + Atezolizumab	Phase 1	NCT02471846

NSCLC, Non-small cell lung cancer; SCLC, Small cell lung cancer; ATL, Adult T-cell leukemia; PTCL, Peripheral T cell lymphoma; TNBC, Triple-negative breast cancer.

#### 4.4.1 Depleting Tregs

##### 4.4.1.1 Targeting Foxp3

The expression of Foxp3 is critical for the development and function of Tregs. However, its overexpression can mediate the activation of Wnt/β-catenin signaling, which promotes tumor growth, metastasis, and epithelial-mesenchymal transition (EMT) (94). Studies have demonstrated that transient genetic depletion of Foxp3 in Tregs can enhance the efficacy of therapeutic vaccines against established melanoma tumors in preclinical models. This indicates that Foxp3 could be a highly attractive target in cancer immunotherapy (95). AZD8701, a first-in-class human clinical candidate antisense oligonucleotide (ASO) inhibitor targeting Foxp3, is currently being evaluated in Phase 1a/b clinical trials (NCT04504669) for its potential use in cancer treatment (96).

##### 4.4.1.2 Targeting IL-2/CD25 signaling

CD25 is predominantly found on Tregs and has a high affinity for IL-2, facilitating the formation of the IL-2/IL-2 receptor complex, which collectively enhances Treg proliferation, survival, and functional integrity. Consequently, CD25 has emerged as a specific target for depleting Tregs in both mouse and human cancers. By altering the IL-2 signaling pathway, CD25 can influence Treg activity. For instance, Daclizumab inhibits the binding of IL-2 to CD25, adversely affecting Tregs (97). Nevertheless, this method has a limited therapeutic range since CD25 is upregulated not only on Tregs but also on T_eff_ cells following activation. On the other hand, the chimeric anti-CD25 antibody basiliximab can prevent T-lymphocyte activation by blocking CD25 subunits on the IL-2 receptor. It has been documented that basiliximab targets CD4^+^CD25^high^ cells while sparing CD4^+^CD25^low^ cells (98).

#### 4.4.2 Targeting immune checkpoints

##### 4.4.2.1 Anti-PD-1/PD-L1

PD-1 is an immune checkpoint receptor extensively found on various immune cells, including T cells, B cells, and NK cells. In T cells, PD-1 predominantly appears on the surface of activated T cells. Its expression in Tregs is essential for maintaining their stability and function, promoting Foxp3 expression and Treg proliferation (99). Additionally, PD-1 suppresses TCR signal transduction in CD8^+^T cells by inhibiting the CD28 co-stimulatory signal, thus preventing overactivation of T_effs_ (100). In the TME, tumor cells and other immunosuppressive cells like macrophages and DCs often overexpress PD-L1. Tumor cells can also boost PD-L1 expression through IFN-γ secretion. The PD-L1/PD-1 interaction reduces T-cell production, cytokine release, and survival, aiding tumors in evading immune surveillance and destruction. Inhibitors targeting PD-1/PD-L1 have become critical therapies for various cancers, with PD-1 inhibitors such as Pembrolizumab showing favorable survival outcomes in patients with urothelial carcinoma (101), non-small cell lung cancer (NSCLC) (102), and melanoma (103). Nivolumab is used in clinical trials for refractory esophagogastric cancer and metastatic small cell lung cancer (SCLC) (104). Atezolizumab, which targets PD-L1, has been approved as a primary monotherapy for metastatic NSCLC patients with high PD-L1 levels and as an adjuvant treatment for resected stage II-IIIA NSCLC patients (105). However, some studies suggest that PD-1 blockade might lead to the expansion of highly suppressive PD-1^+^ eTregs, which can hinder anti-tumor immune responses, indicating limitations in monotherapy (106). Therefore, combining PD-1/PD-L1 inhibitors with other treatments like radiotherapy, chemotherapy, CTLA-4 inhibitors, or tumor vaccines may offer improved therapeutic outcomes.

##### 4.4.2.2 Anti-CTLA-4

CTLA-4 plays a vital role in sustaining the immunosuppressive functions of Tregs through its constant expression on these cells. In mouse models, blocking CTLA-4 *in vivo* can trigger autoimmune diseases and disrupt the inhibitory effects mediated by Tregs *in vitro* (107). This underscores the essential role of CTLA-4-expressing Tregs in mediating immune suppression. Mechanistically, Tregs control the levels of CD80/CD86 on the surfaces of APCs via a CTLA-4-dependent pathway, preventing CD28 from binding to CD80/CD86 and thereby hindering the signaling necessary for T-cell proliferation. Besides interacting with CTLA-4 on T cells, CD80 also engages with PD-L1 on APCs. Studies have shown that reducing CD80/CD86 levels leads to an increase in PD-L1 on APCs, enabling Tregs to suppress not only naïve T cells but also T_eff_ cells expressing PD-1 (108). Monoclonal antibodies (mAbs) targeting CTLA-4 have revolutionized cancer treatment by inducing lasting tumor regression, although their mechanisms of action are not fully understood. Initially, it was thought that anti-CTLA-4 antibodies worked by blocking CTLA-4, thereby enhancing the binding between CD28 and CD80/CD86 and reducing T-cell inhibition (109). However, recent findings suggest that these antibodies may also mediate antibody-dependent cellular cytotoxicity/phagocytosis (ADCC/P), leading to Treg depletion and enhancing CD8^+^T cell responses specific to tumor antigens (110). Additionally, CTLA-4 mAbs can activate myeloid cells and modify the TME through interactions with FcγR (111). These insights offer new perspectives on the mechanisms of therapies targeting CTLA-4. Clinically, combining PD-1 and CTLA-4 immune checkpoint inhibitors (ICIs) has significantly improved survival outcomes in patients with melanoma, highlighting the benefits of combination therapy (112).

##### 4.4.2.3 Targeting other immune checkpoints

LAG-3, TIM-3, and TIGIT are inhibitory receptors found on Tregs, presenting new targets for immunotherapy. LAG-3 negatively regulates T cell function and mediates bidirectional signaling in APCs (113). During interactions between Tregs and DCs, the engagement of LAG-3 on Tregs enhances their activity, promoting immune tolerance and indirectly inhibiting DC function. Furthermore, the interaction of LAG-3 with its ligand, MHC class II, can downregulate cytokine secretion and proliferation of CD4^+^T cells (113). Current research is exploring anti-LAG-3 antibodies both alone and in combination with other immunotherapies for treating melanoma. Compared to LAG-3 blockade alone, the combination of LAG-3 and PD-1 inhibitors has shown a more promising response rate in patients with refractory melanoma (114). Relatlimab combined with Nivolumab as a first-line treatment for metastatic melanoma patients has demonstrated improved progression-free survival compared to PD-1 blockade alone (115). Favezelimab, an emerging mAb targeting LAG-3, has shown significant efficacy when combined with pembrolizumab in treating patients with advanced microsatellite-stable CRC (116).

Studies have demonstrated that TIM-3^+^Tregs express higher levels of cell markers such as FoxP3, LAG-3, and CTLA-4, indicating significantly enhanced suppressive functions. These Tregs specifically inhibit Th17 cells, highlighting the potential value of TIM-3 as a target for cancer therapy (117). Similarly, Phase I studies of the anti-TIM-3 antibody Sabatolimab, used either as a monotherapy or in various combinations (including with PD-1 inhibitors), are being conducted in patients with advanced solid tumors. Early results indicate that the combination of Sabatolimab and Spartalizumab is well-tolerated and exhibits anti-tumor activity (118).

Additionally, TIGIT is highly expressed on Tregs and suppresses Th1 and Th17 cells through the secretion of Fgl2. It may also contribute to the generation of tolerogenic DCs, thereby inhibiting the activation of T_eff_ responses (119).Therapeutic strategies targeting TIGIT involve receptor-ligand blockade independent of Fc interactions, clearance of TIGIT-expressing Tregs through Fc-mediated mechanisms, and Fc-dependent modulation of myeloid cells (120). A clinical trial has revealed that the objective response rate (ORR) for NSCLC patients treated with both the anti-TIGIT antibody tiragolumab and atezolizumab is 31%, compared to 16% with atezolizumab monotherapy, highlighting the superior clinical benefits of combination therapy (120).

#### 4.4.3 Targeting other co-stimulation molecules

Anti-tumor immunity can also be achieved by activating co-stimulatory receptors on T cells using agonistic antibodies. These co-stimulatory receptors, mainly from the tumor necrosis factor receptor superfamily (TNFRSF), include OX40, ICOS, and GITR. OX40 is a co-stimulatory molecule constantly expressed by Tregs and induced in activated T_effs_. In an open-label phase I/II study, the anti-OX40 antibody, including the mAb INCAGN01949, is being assessed for safety and pharmacodynamics in treating advanced malignancies (NCT02923349) (121). Another novel anti-OX40 antibody, BAT6026, which has enhanced antibody-dependent cell cytotoxicity, facilitates intra-tumoral Treg depletion and is being utilized in cancer immunotherapy (122).

Therapeutic antibodies targeting GITR have shown therapeutic efficacy in preclinical tumor models, characterized by reduced and functionally altered intra-tumoral Tregs and enhanced anti-tumor CD8^+^T cell function (123). Currently, studies are ongoing to evaluate GITR agonists either alone or in combination therapies for patients with advanced solid tumors (NCT02583165 and NCT02628574) (124). The GITR agonist TRX518, both as a monotherapy and in combination with pembrolizumab or nivolumab, has demonstrated acceptable safety in phase I studies (125).

Vopratelimab, an ICOS agonist has shown good safety profiles in patients with advanced solid tumors, both as a monotherapy and in combination with nivolumab (NCT02904226). This therapy upregulates ICOS expression on CD4^+^T cells after T-cell priming, enhancing their proliferation and activation (126).

#### 4.4.4 Targeting Treg cytokine secretion

The inhibition of the TGF-β pathway is garnering significant attention in cancer research. Currently, antibodies targeting TGF-β are categorized into two main types: those directly inhibiting TGF-β activity, such as neutralizing antibodies like Fresolimumab, SAR439459, and NIS793, and those targeting TGF-β receptors, including LY3200882, Vactosertib, and Galunisertib. These inhibitors can block TGF-β signaling pathways, with some already in clinical trials. For example, Fresolimumab has been assessed for its efficacy in patients with late-stage melanoma or renal cell carcinoma (127). Vactosertib in conjunction with Durvalumab is under examination for treating gastric cancer (NCT04893252), while its combination with Pembrolizumab is being tested for advanced CRC or gastric cancer (NCT03724851). Similarly, Galunisertib paired with Nivolumab is being assessed for NSCLC or hepatocellular carcinoma. Furthermore, therapies that concurrently target TGF-β and PD-L1 pathways have yielded promising results. Bintrafusp alfa, a bifunctional fusion protein that inhibits TGF-βRII and blocks PD-L1, has shown positive clinical outcomes in second-line therapy for NSCLC and first-line treatment for cervical cancer patients (128).

IL-10 and IL-35 play crucial roles in modulating the immune response to tumors. IL-10 supports Treg stability and phenotype by maintaining Foxp3 and TGF-β expression and influences Treg cell differentiation by inhibiting Akt phosphorylation and preserving Foxo1 function (129). IL-35 is overexpressed in various cancers, directly correlating with tumor progression and poor prognosis. Treg cell-derived IL-10 and IL-35 induce exhaustion in CD8^+^T cells by upregulating the expression of BLIMP-1 and genes associated with exhaustion, leading to CD8^+^T cell dysfunction (130). Neutralizing antibodies against IL-35 not only enhance gemcitabine chemotherapy but also significantly reduce microvascular density in mouse models of pancreatic cancer (131). Therefore, targeting IL-35 within the TME could provide a promising approach for tumor immunotherapy.

#### 4.4.5 Inhibiting Treg migration

Another intriguing aspect of Tregs is their elevated expression of chemokine receptors CCR4 and CCR8, which presents exciting therapeutic opportunities. The CCR4 mAb KM2760 was originally employed to treat adult T-cell leukemia/lymphoma (ATLL). This antibody boosts ADCC by enhancing its affinity for FcγR on effector cells, aiming to reduce Treg numbers and initiate a potent anti-tumor immune response (132). Although Mogamulizumab, in combination with Nivolumab, does not show increased efficacy in a phase I/II study for advanced solid tumors, it does exhibit promising safety and tolerability profiles (133). Unlike Mogamulizumab, anti-CCR8 antibodies can selectively target and deplete Tregs, thereby enhancing immune responses against tumors (134). Additionally, research suggests that combining CCR8 targeting with anti-PD-1 therapy could effectively eliminate TI-Tregs (135). These findings open up new avenues for targeting Tregs via CCR4 and CCR8, positioning them as pivotal players in the future of tumor immunotherapy.

#### 4.4.6 Targeting Tregs metabolism

Tumor metabolism can significantly impede the efficacy of immunotherapy, particularly through the actions of TI-Tregs. Therefore, targeting the metabolism of these Tregs holds considerable immunological and metabolic promise and could be a strategy to overcome metabolic barriers in immunotherapy. The Phosphoinositide 3-kinase/protein kinase B/mammalian target of rapamycin (PI3K/AKT/mTOR) pathway not only regulates the expression of Foxp3 in Tregs but also plays a vital role in key metabolic processes like glycolysis (136).

Alpelisib, the pioneering PI3Kα inhibitor used for breast cancer treatment, has extended progression-free survival in patients with HR^+^/HER2^-^ metastatic breast cancer carrying PIK3CA mutations when combined with Fulvestrant (NCT02437318) (137). Additionally, Alpelisib in combination with Olaparib has shown good tolerability in pre-treated triple-negative breast cancer (TNBC) patients (NCT01623349) (138). The PI3Kδ inhibitor Idelalisib is approved for treating B-cell malignancies (NCT01539512). Duvelisib, a PI3K-δ/γ inhibitor, has demonstrated clinical activity and safety in T-cell lymphomas (NCT01476657). Gedatolisib, which selectively targets all class I PI3K subtypes (p110α, p110β, p110γ, and p110δ) and mTOR, significantly avoids resistance development. By targeting these class I PI3K and mTOR, Gedatolisib has shown promising ORR in breast cancer treatment (NCT02684032) (139).

mTOR activity is often upregulated in human cancers. Combining Everolimus with hormone therapy has improved progression-free survival in patients with advanced HR^+^ breast cancer (NCT00863655), and it has also demonstrated good tolerability in treating metastatic pancreatic cancer patients (NCT00409292) (140). IDO, the primary enzyme in tryptophan (Trp) degradation metabolism, converts Trp into kynurenine (Kyn). Overexpression of IDO and the resulting accumulation of Kyn in tumors can inhibit T_eff_ cells and activate Tregs by binding to the aryl hydrocarbon receptor (AHR) (141). Although several IDO1 inhibitors, including INCB024360, GDC-0919, and NLG802, are not yet approved by the US FDA for cancer therapy, some have advanced to clinical trials. For instance, the combination of Navoximod (GDC-0919) and the PD-L1 inhibitor Atezolizumab has shown acceptable safety and tolerability in patients with late-stage cancer (NCT02471846) (142). Further clinical investigation is necessary to target IDO as a blockade point, and exploring other targets related to amino acid metabolism remains to be an illustrated area.

## 5 Conclusion

Tregs, crucial regulators of immune homeostasis, are of significant interest in both autoimmune and anti-tumor immune clinical settings. Therapies targeting Tregs are currently being actively explored, either as standalone treatments or in combination with other therapeutic approaches such as immune checkpoint blockade, targeted vaccine therapy, radiotherapy, and chemotherapy, especially when combined with ICIs. Strategies to increase the number of Tregs can be applied in treating autoimmune conditions and other disorders requiring immunosuppression. Conversely, depleting Tregs holds great potential in cancer treatment, although it may lead to harmful autoimmune reactions. Therefore, targeting terminally differentiated eTregs rather than all Foxp3^+^T cells, is a crucial strategy. For instance, CTLA-4 inhibitors can either eliminate eTregs or diminish their suppressive function (143). Overall, despite some clinical success in targeting Tregs, a deeper understanding of their formation, maintenance, and function is crucial to improve precision-targeted immunotherapy against Tregs.
